# Association of physical activity and PM2.5-attributable cardiovascular disease mortality in the United States

**DOI:** 10.3389/fpubh.2023.1224338

**Published:** 2023-09-28

**Authors:** Yingying Liu, Mengmeng Yan

**Affiliations:** ^1^Department of Health Management and Institute of Health Management, Sichuan Provincial People's Hospital, University of Electronic Science and Technology of China, Chengdu, China; ^2^Chinese Academy of Sciences Sichuan Translational Medicine Research Hospital, Chengdu, China; ^3^School of Healthcare and Technology, Chengdu Neusoft University, Chengdu, China

**Keywords:** PM2.5, cardiovascular deaths and mortality, physical activity, United States, cardiovascular diseases

## Abstract

**Objective:**

The study aimed to explore the association between physical activity (PA) and PM2. 5-attributable cardiovascular disease (CVD) mortality trends across the United States (US) at the state level.

**Methods:**

We conducted a cross-sectional study using data from the Global Burden of Disease 2019 study for PM2.5-attributable CVD mortality and the Behavioral Risk Factor Surveillance System for PA prevalence. The study covered all 50 US states and the District of Columbia from 2001 to 2019. We utilized Joinpoint Regression to calculate AAPC from 2011 to 2019 and Pearson correlation coefficients to assess state-level associations between PA and PM2.5-attributable CVD mortality AAPC.

**Results:**

During 2011–2019, a total of 244,318 PM2.5-attributable CVD deaths were recorded. The age-adjusted mortality rates (AAMR) of PM2.5-attributable CVD declined substantially from 2011 to 2019 across all US states, with the most pronounced reductions observed in industrialized states such as West Virginia (51% decline), Kentucky (32%), and Ohio (22%). AAMR ratios for the US states varied substantially, ranging from 0.1 in Hawaii to 1.7 in Arkansas. The AAPC ranged from −9.4% (West Virginia) to −1.7% (New Mexico) in the majority of states, while a few states such as Alaska, Wyoming, and Washington saw slight positive AAPCs from 0.9 to 2.9%. A significant correlation was found between PA and PM2.5-attributable CVD mortality trends (*r* = 0.454, *p* = 0.001), with similar results in subgroup analyses.

**Conclusion:**

Our findings suggest a correlation between increased physical activity (PA) and increased PM2.5-attributable CVD mortality, highlighting the potential need to consider PM2.5 exposure when engaging in PA to mitigate adverse cardiovascular health impacts. However, further research is warranted to establish causality and underlying mechanisms in the relationship between PA and PM2.5-attributable CVD mortality. Potential limitations include reliance on self-reported PA data.

## 1. Introduction

Air pollution, particularly fine particulate matter (PM2.5), has been identified as a major contributor to cardiovascular disease (CVD) mortality worldwide. In the United States (US) alone, PM2.5 exposure has been linked to ~100,000 premature deaths annually, with nearly 25% attributable to CVDs (1, 2). Specifically, national estimates indicate that CVD mortality rates attributable to PM2.5 are the highest in the Midwest and Southern regions of the US, with rates exceeding 60 deaths per 100,000 population in states such as Arkansas and Oklahoma (3). In 2019 (2), there were significant variations in PM2.5-attributable CVD mortality rates among different countries. For instance, Germany had a PM2.5-attributable CVD mortality rate of 18.84 per 100,000 population for all ages, while Australasia had a rate of 3.79, the United Kingdom had 10.58, the US had 7.84, Japan had 15.53, France had 9.69, and Canada had 5.13. It is worth noting that after age-standardization, the data still showed similar disparities. For age-standardized rates, Germany had a PM2.5-attributable CVD mortality rate of 7.54 per 100,000 population, Australasia had 2.08, the United Kingdom had 5.38, the US had 4.53, Japan had 5.01, France had 4.10, and Canada had 2.61. These significant PM2.5-related health burdens underscore the need to identify modifiable factors that may mitigate the cardiovascular toxicity of air pollution.

Air pollution arises from various sources, including both anthropogenic (human-made) and natural sources. Anthropogenic sources include emissions from industries, transportation (e.g., vehicles, airplanes, and ships), power generation, and residential activities (e.g., cooking and heating). These sources release a complex mixture of pollutants, including PM2.5, nitrogen oxides (NOx), sulfur dioxide (SO_2_), volatile organic compounds (VOCs), and carbon monoxide (CO). Industrial activities, such as manufacturing and combustion of fossil fuels, are major contributors to PM2.5 emissions. Transportation-related emissions also play a significant role, especially in urban areas with high vehicle density. Combustion of fossil fuels in power plants for electricity generation is another important source of PM2.5 and other air pollutants. Additionally, residential activities, such as burning wood and other solid fuels for heating and cooking, contribute to PM2.5 levels, particularly in rural and developing areas. Natural sources of PM2.5 include wildfires, volcanic eruptions, dust storms, and pollen release. While these events can contribute to localized increases in PM2.5 levels, they generally do not contribute as significantly to long-term exposure as anthropogenic sources.

Physical activity (PA) has been well-established as a protective factor against CVDs and related mortality (4, 5). However, most studies demonstrating the cardiovascular benefits of PA have been conducted in areas with relatively low air pollution levels. Emerging evidence suggests that engaging in outdoor PA in polluted environments may limit the cardioprotective effects of exercise (6). Several potential mechanisms have been proposed, including enhanced pulmonary inflammation, elevated blood pressure, and increased deposition of particles in the lungs during exercise (7). Nevertheless, few studies have directly investigated the complex interplay between PM2.5 and PA on a population level.

While some research studies have reported that outdoor PA may exacerbate PM2.5-attributable CVD risks (8, 9), other studies have suggested that PA benefits may outweigh risks across all levels of PM2.5 exposures (10). However, existing studies have been limited to specific cohorts, providing inadequate evidence to guide public health policies on balancing the benefits and risks of promoting PA in polluted areas. Our study aimed to address this critical research gap by leveraging nationwide data to examine the association between state-level PA rates and ambient PM2.5-attributable CVD mortality across the US. Elucidating the interacting effects between PA and PM2.5 is crucial to inform air quality regulations and tailored interventions that maximize the cardiovascular benefits of PA while minimizing risks of air pollution exposures. Findings from this state-level analysis will provide novel insights unavailable from individual-level data, helping guide targeted policies and public health strategies to address the substantial spatial disparities in PM2.5-attributable CVD mortality across the US (11).

## 2. Materials and methods

This study follows the Strengthening the Reporting of Observational Studies in Epidemiology (STROBE) reporting guidelines for cross-sectional studies.

### 2.1. PM2.5-attributable cardiovascular diseases data

We used the Global Health Data Exchange (2) to extract the data on cardiovascular disease mortality attributable to PM2.5 in the US from 1990 to 2019. The PM2.5-attributable cardiovascular disease data in the US included sex, states, cardiovascular disease subtypes, and age-adjusted mortality rates (AAMR). Exposure to PM2.5 is defined as the population-weighted annual average mass concentration of particles with an aerodynamic diameter of <2.5 μm in a cubic meter of air.

To assess the burden attributable to particulate matter, exposure levels were evaluated for every country globally. PM2.5 exposures were estimated using satellite data, ground measurements, and chemical transport models applied to a grid network. These grid-level exposure estimates were then weighted by the estimated population in each grid cell to derive national average exposure levels. Country-specific estimates and uncertainty intervals (95% uncertainty) for ambient PM2.5 exposure were generated by taking 1,000 draws of each grid cell value. After estimating PM2.5 exposures, attributable risks were calculated using the GBD integrated exposure-response model, which predicts the relative risk of mortality according to annual average PM2.5 levels based on risk estimates from studies of outdoor air pollution. By adjusting for median age, mortality estimates were rescaled to derive incidence rates. Details on the specific methodology can be found in appendix 1 of the GBD study (12).

Cardiovascular diseases include ischemic heart diseases (International Classification of Diseases, 10th revision code I20–I21.6, I21.9–I25.9, Z82.4–Z82.49) and stroke (International Classification of Diseases, 10th revision code G45–G46.8, I60–I62, I62.9–I64, I64.1, I65–I69.998, Z82.3).

### 2.2. PA data

PA data were directly obtained from the Behavioral Risk Factor Surveillance System (BRFSS) (13). The BRFSS defined PA as participation in ≥150 min of aerobic activity per week, which was calculated based on responses to one or more survey questions (13). The standardized survey questions have demonstrated strong reliability and validity (14). The PA data of the US included sex, state, age, and age-adjusted prevalence.

### 2.3. Statistics

We used the Joinpoint Regression statistical software (National Cancer Institute, version 4.9.0.0) to calculate the average annual percent change (AAPC) from 2011 to 2019 because the earliest available PA data were from 2011, while the Global Burden of Disease (GBD) data on cardiovascular mortality was up to 2019. We used log transformation and identified up to two inflection points. Annual percent change characterizes trends in AAMR over time, and AAPC describes the average annual percent change over a period of multiple years. Pearson correlation coefficients were used to test the state-level correlations between PA and the AAPC of PM2.5-attributable CVD mortality. A two-sided *p*-value of < 0.05 was considered to be statistically significant. Data were analyzed from 15 November 2022 to 15 December 2022.

### 2.4. Sensitivity analysis

In the sensitivity analysis, we extracted the prevalence data for muscle-strengthening activities (MSA) from BRFSS to replace the PA prevalence and redo the analysis. MSA is defined as participating in muscle-strengthening exercises two or more times per week.

## 3. Results

### 3.1. PM2.5-attributable CVD mortality rates and trends in the US

During 2011–2019, there were a total of 244,318 PM2.5-attributable deaths related to CVD, with a mean AAMR of 5.22 (95% CI: 3.02–7.53) per 100,000 person-years. Male patients were found to be more vulnerable (Table 1). Compared to the period 2002–2010 (AAMR: 5.36, 95% CI: 9.55–13.94), there has been a decrease in the PM2.5-attributable CVD mortality rate during 2011–2019. The AAMR of PM2.5-attributable CVD decreased nationally with an AAPC of −4.1% (−4.6, −3.7) per year over the study period (Table 1). Similar trends were seen across subgroups including subtypes and sex (Table 1). Ischemic heart disease has a higher AAMR than stroke.

**Table 1:** Age-adjusted PM2.5-attributable mortality rates and Joinpoint trends in the US by sex and subtypes during 2011–2019.

**Characteristics**	**No. of patients**	**Mean AAMR**	**AAPC**
CVD	244,318	5.22	−4.1 (−4.6, −3.7)
Male	134,655	6.68	−4.5 (−5.1, −3.9)
Female	109,662	3.94	−3.69 (−4.2, −3.6)
IS	182,529	3.90	−4.2 (−4.6, −3.9)
Male	107,034	5.30	−4 (−4.3, −3.7)
Female	75,495	2.97	−4.8 (−5.4, −4.2)
Stroke	61,788	1.31	−3.8 (−4.3, −3.4)
Male	27,620	1.38	−4 (−4.6, −3.4)
Female	34,167	1.24	−3.6 (−3.9, −3.3)

IS, ischemic heart disease, AAMR, age-adjusted mortality rates, AAPC, average annual percent change. All the p-values of AAPC are < 0.05.

### 3.2. State-level disparity in PM2.5-attributable CVD mortality rates and trends

Table 2 demonstrates the state-level AAMR of PM2.5-attributable CVD during 2011–2019. In 2011, CVD AAMR were the highest in Arkansas (10.7, 95% CI: 7.2, 14.1) and the lowest in Wyoming (0.93, 95% CI: 0.1, 2.6), with a mortality rate ratio of 11.5 between the highest and lowest mortality states. The state-level AAMR of PM2.5-attributable CVD showed overall downward trends from 2011 to 2019, indicative of substantial progress in PM2.5 pollution control nationwide. The most pronounced decline was observed in West Virginia, where the AAMR decreased by 51% from 8.25 in 2011 to 4.00 in 2019. Several industrialized states in the East and Midwest regions, including Kentucky (32% decline), Ohio (22%), and Indiana (26%), also experienced large AAMR reductions. In contrast, the decrease was more modest in some Western states such as Wyoming, Hawaii, and New Mexico, which had relatively lower baseline AAMRs in 2011. A few states such as Alaska and Montana saw a slight AAMR increase though the magnitudes were minimal. Between 2011 and 2019, AAMR decreased in most states but increased in Alaska with an AAPC of 2.9 (2.0–3.9). The AAMR ratios (MRRs) for American states vs. America varied substantially from 0.1 in Hawaii to 1.7 in Arkansas (Table 2). A total of 22 states had an MRR of more than 1.0.

**Table 2:** State-level AAMR of PM2.5-attributable cardiovascular diseases during 2011–2019.

**States**	**AAMR 2011**	**AAMR 2019**	**Mean AAMR**
Alabama	10.57	6.65	8.14
Alaska	3.72	4.63	3.48
Arizona	3.72	3.19	3.37
Arkansas	10.69	7.51	8.69
California	6.22	5.15	5.48
Colorado	2.66	2.13	2.20
Connecticut	3.53	2.27	2.73
Delaware	7.25	4.55	5.49
District of Columbia	10.08	5.66	7.61
Florida	4.30	2.44	3.26
Georgia	9.01	6.25	7.08
Hawaii	1.17	0.71	0.74
Idaho	2.76	3.01	3.16
Illinois	7.77	5.56	6.47
Indiana	9.22	6.84	7.80
Iowa	5.97	4.51	5.12
Kansas	5.85	4.01	4.74
Kentucky	9.53	6.44	7.58
Louisiana	8.47	6.07	7.02
Maine	1.85	1.03	1.34
Maryland	7.27	4.67	5.64
Massachusetts	3.10	1.73	2.22
Michigan	6.35	5.38	5.81
Minnesota	3.10	2.32	2.68
Mississippi	10.24	6.58	8.08
Missouri	8.66	5.81	6.89
Montana	2.38	2.55	2.87
Nebraska	4.91	3.34	3.97
New Hampshire	2.39	1.43	1.68
New Jersey	5.75	3.96	4.53
New Mexico	1.87	1.62	1.69
New York	5.18	3.50	4.10
North Carolina	6.96	4.42	5.27
North Dakota	2.91	2.41	2.74
Ohio	8.46	6.60	7.36
Oklahoma	10.02	7.18	8.11
Oregon	2.77	2.64	2.82
Pennsylvania	7.88	5.63	6.38
Rhode Island	4.15	2.36	3.11
South Carolina	8.07	5.30	6.24
South Dakota	3.51	2.88	3.16
Tennessee	9.24	5.83	7.16
Texas	8.33	6.05	7.00
Vermont	2.04	1.17	1.44
Virginia	5.88	3.36	4.34
Washington	2.45	2.23	2.47
West Virginia	8.25	4.00	6.23
Wisconsin	5.13	4.17	4.56
Wyoming	0.93	0.90	0.89

AAMR, age-adjusted mortality rates; CI, 95% confidence intervals.

**Figure 1:**
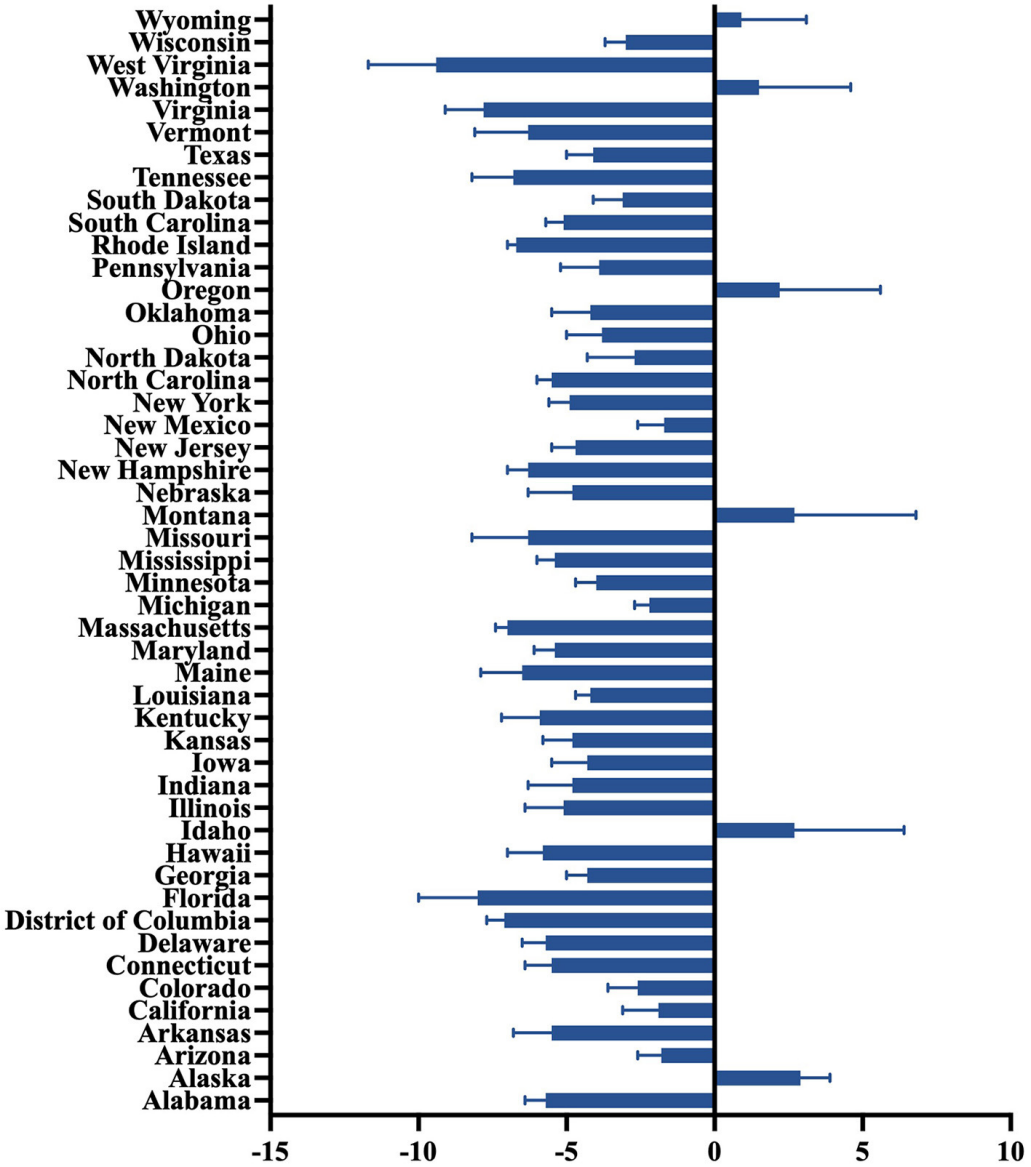
State-level average annual percent change of PM2.5-attributable cardiovascular diseases during 2011–2019.

Figure 1 showed predominant downward trends across most US states from 2011 to 2019, indicative of substantial progress in mitigating PM2.5 pollution and associated public health risks. Predominant downward trends were observed, with the AAPC ranging from −9.4% (West Virginia, 95% CI: −11.7 to −7.1%) to −1.7% (New Mexico, 95% CI: −2.6 to −0.8%) in the majority of states. Some states, such as Alaska (AAPC: 2.9%, 95% CI: 2.0 to 3.9%), Wyoming (AAPC: 0.9%, 95% CI: −1.2 to 3.1%), and Washington (AAPC: 1.5%, 95% CI: −1.5 to 4.6%), saw slight positive AAPCs. Notably, West Virginia (−9.4%), Virginia (−7.8%, 95% CI: −9.1 to −6.5%), Massachusetts (−7.0%, 95% CI: −7.4 to −6.6%), and Rhode Island (−6.7%, 95% CI: −7.0 to −6.4%) exhibited the steepest AAPC declines. Additionally, industrialized states in the Midwest and East, such as Kentucky (−5.9%, 95% CI: −7.2 to −4.5%), Ohio (−3.8%, 95% CI: −5.0 to −2.5%), and Indiana (−4.8%, 95% CI: −6.3 to −3.3%), showed relatively large reductions. According to AAPC of America, 17 states saw a slower decline in velocity (Figure 1).

### 3.3. Ecological correlations between physical activity and PM2.5-attributable CVD mortality trends

State-level age-adjusted prevalence of physical activity remained overall stable between 2011 (mean, 51.0%; range, 33.9%−61.9%) and 2019 (mean, 49.9%; range, 30.4%−62.4%). It was the highest in Colorado (mean, 59.9%) and the lowest in Mississippi (mean, 40.1%) in 2011–2019. Physical activity levels were relatively similar among the male subgroup (mean crude prevalence, 51.6%; 95% CI, 49.0%−54.2%) and female subgroup (mean crude prevalence, 49.2%; 95%CI, 46.9%−51.5%).

**Figure 2:**
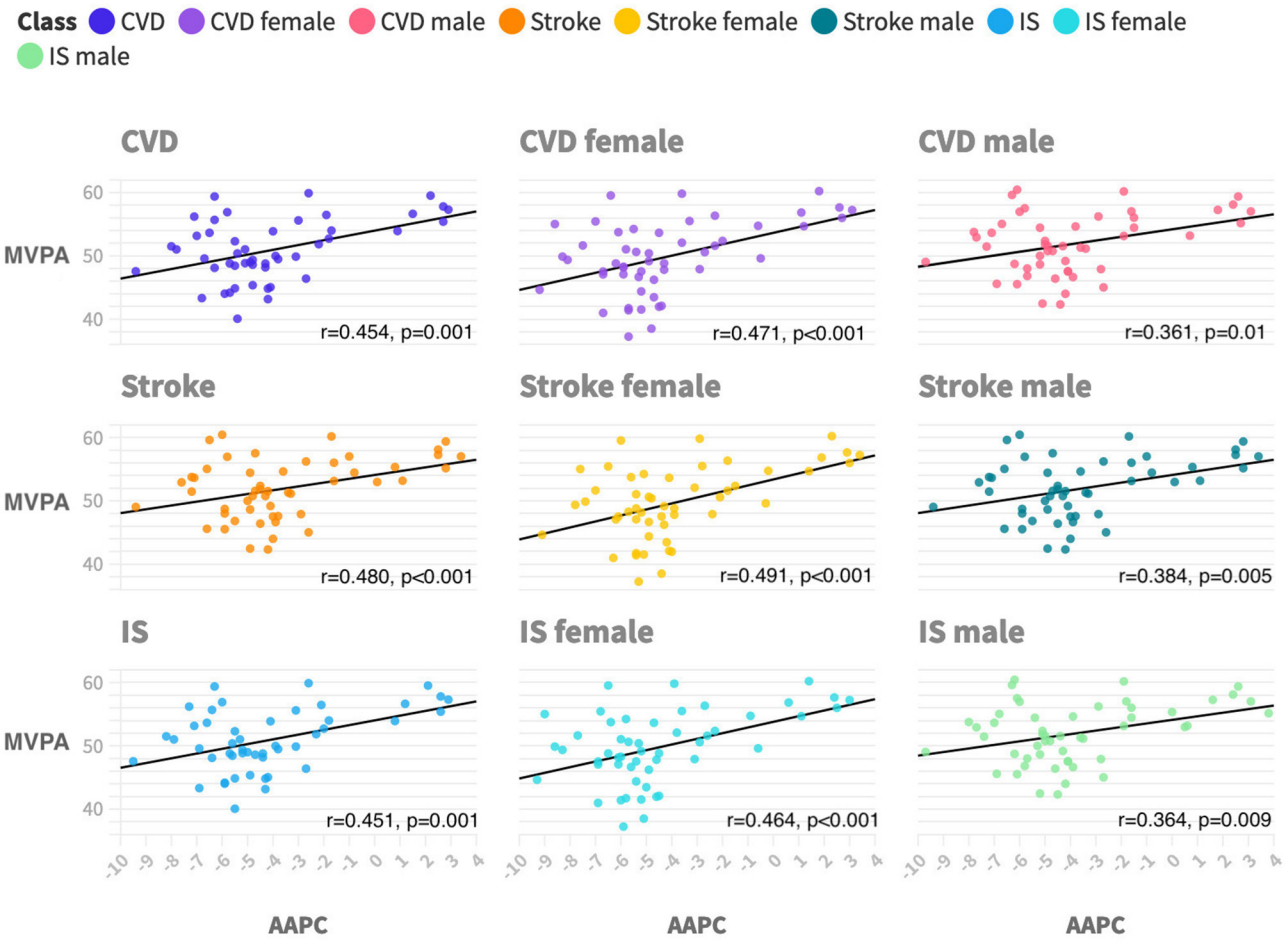
Ecological correlations between physical activity and PM2.5-attributable CVD mortality trends by subtypes and sex during 2011 to 2019. CVD is cardiovascular disease. IS is ischemic heart disease. MVPA is moderate-to-vigorous aerobic physical activity, which is defined as participating in 150 min or more of exercise each week. AAPC is average annual percentage changes. All *p*-values are < 0.05 with a two-tailed test.

Figure 2 shows that the AAPC of PM2.5-attributable CVD mortality in each state had a moderate positive correlation with state-level physical activity (*r* = 0.454, *p* = 0.001). Similar associations were seen across subtypes including IS (*r* = 0.451, *p* = 0.001) and stroke (*r* = 0.48, *p* < 0.001). Similar associations were also seen across sex subgroups, and the correlation coefficient between PA and female patients is greater than that of male patients.

### 3.4. Sensitivity analysis

State-level age-adjusted prevalence of MSA increased between 2011 (mean, 29.2%; range, 27.6%−30.9%) and 2019 (mean, 35.1%; range, 33.3%−36.9%). It was the highest in Colorado (mean, 36.7%) and the lowest in West Virginia (mean, 23.3%) from 2011 to 2019. State-level MSA prevalence positively correlated with PM2.5-attributable CVD mortality trend (*r* = 0.36, *p* = 0.010), PM2.5-attributable IS mortality trend (*r* = 0.33, *p* < 0.05), and PM2.5-attributable stroke mortality trend (*r* = 0.37, *p* < 0.005). However, in the sex subgroup, there is a significant positive correlation between MSA and mortality trends in female patients, while there was no significant correlation between MSA and AAPC in male patients, regardless of CVD or its subtypes.

## 4. Discussion

In this study, we observed considerable state-level disparities in trends of AAMR for PM2.5-attributable CVD, which is associated with PA. However, it is crucial to note that the cross-sectional design of the study limits our ability to establish causality definitively. The observed association between PA and PM2.5-attributable CVD mortality may be influenced by various confounding factors that were not adequately addressed in the study. Therefore, caution should be exercised when interpreting the findings as a direct causal relationship.

Our results suggested that the cardiovascular benefits of PA might be reduced, offset, or even reversed by PM2.5 exposure. The detrimental impact of PM2.5 on the cardiovascular system may occur through several pathways (15): (1) Increased inhalation of pollutants: engaging in PA, particularly vigorous exercise, leads to increased ventilation and deeper breathing, which can result in a higher intake of air pollutants, including PM2.5 (16). This increased exposure may exacerbate the detrimental effects of air pollution on cardiovascular health. (2) Oxidative stress and inflammation: PM2.5 exposure has been associated with increased oxidative stress and systemic inflammation, both of which are known risk factors for CVD (17, 18). PA, especially when performed in polluted environments, may exacerbate these harmful effects, potentially negating the cardiovascular benefits of exercise. (3) Endothelial dysfunction: PM2.5 exposure can cause endothelial dysfunction, a critical factor in the development of atherosclerosis and CVD (19–22). Physical activity in polluted environments may further impair endothelial function, offsetting the protective effects of exercise on the cardiovascular system. (4) Autonomic nervous system imbalance: PM2.5 exposure has been linked to autonomic nervous system imbalance, which can lead to an increased risk of cardiac events (4). Engaging in PA in areas with high PM2.5 levels may aggravate this imbalance, potentially reversing the cardiovascular benefits of exercise.

**Figure 3:**
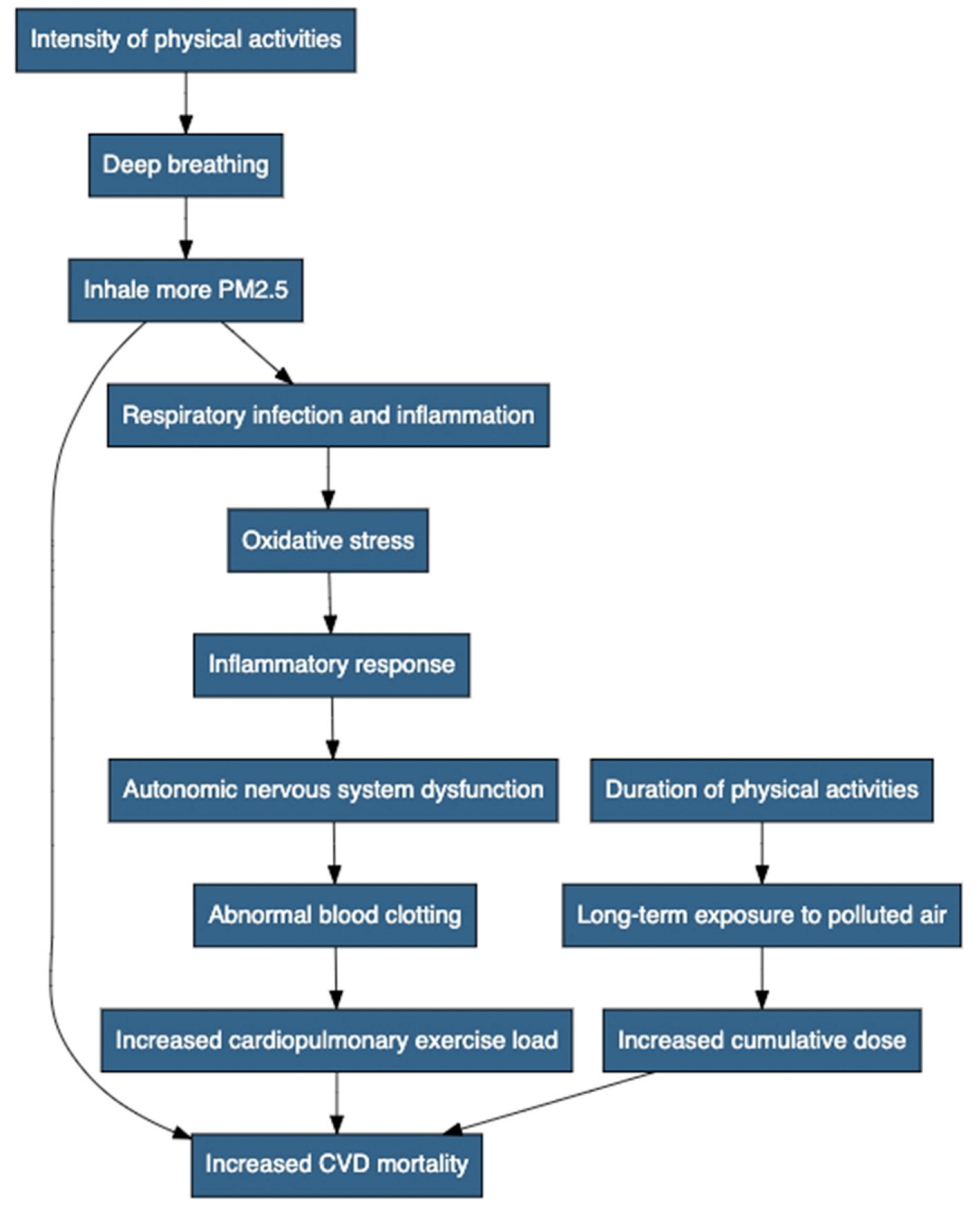
Negative impact mechanisms of physical activity on cardiovascular disease mortality caused by PM2.5. It includes the following steps: intensity of PA, deep breathing, inhaling more PM2.5, increased CVD mortality, duration of PA, long-term exposure to polluted air, increased cumulative dose, respiratory infection and inflammation, oxidative stress, inflammatory response, autonomic nervous system dysfunction, abnormal blood clotting, and increased cardiopulmonary exercise load.

The intensity of PA can play a crucial role in determining the extent to which individuals are exposed to PM2.5 (Figure 3). High-intensity activities that involve heavy breathing, such as running and intense aerobic exercises, may lead to deeper inhalation of polluted air, resulting in an elevated intake of PM2.5 particles. Consequently, individuals participating in high-intensity PA in areas with high PM2.5 levels may face an increased risk of PM2.5-related CVD mortality compared to those engaging in low-to-moderate intensity activities. The duration of PA is another important factor influencing the PM2.5 exposure level. Prolonged exposure to polluted air during extended sports sessions may lead to a higher cumulative dose of PM2.5, potentially amplifying the cardiovascular health risks associated with air pollution. Individuals participating in lengthy outdoor sports events, such as marathons or cycling races, in areas with high PM2.5 concentrations may face a more substantial burden of PM2.5-attributable CVD mortality compared to those engaging in shorter-duration activities.

In Table 2, we observed significant disparities in PM2.5-attributable CVD mortality rates among different states from 2011 to 2019. These disparities may be influenced by various factors, including differences in air pollution levels, socioeconomic conditions, population characteristics, and lifestyle factors among the states. More polluted states such as Arkansas might experience higher PM2.5 exposure, which can contribute to increased CVD mortality rates. Conversely, states with lower pollution levels, such as Wyoming, may have a lower burden of PM2.5-related CVD deaths.

The observed trends in PM2.5-attributable CVD mortality rates from 2011 to 2019 have significant implications for public health. The overall decrease in the national AAMR by −4.1% (95% CI: −4.6% to −3.7%) reflects progress in mitigating PM2.5 pollution and associated cardiovascular health risks. These findings align with previous studies that have demonstrated the effectiveness of air quality improvement measures and their positive impact on reducing CVD mortality rates (12).

State-level disparities in PM2.5-attributable CVD mortality rates are crucial to consider when formulating targeted interventions. The variation in mortality rates among states suggests the need for tailored strategies addressing unique local challenges. High mortality states, such as Arkansas, may require enhanced efforts to control air pollution sources and improve healthcare access to reduce the CVD burden. On the other hand, states with lower mortality rates, such as Wyoming, could benefit from maintaining and reinforcing successful pollution control measures to sustain their positive health outcomes.

Comparing our results to previous studies highlights the consistency of our findings with broader trends (18). The downward trends in most states echo national-level progress in mitigating PM2.5 pollution. The observed positive AAPCs in a few states emphasize the importance of continuous monitoring and targeted interventions in regions experiencing slower declines.

Our findings also align with two previous large-scale studies (23, 24). In a national study of young South Koreans, the benefits of PA were diminished in highly polluted environments (25). Similarly, a survey of six low- and middle-income countries found that high PA increased the risk of stroke attributable to PM2.5 (26). Our findings further demonstrate a positive association between PA and PM2.5-attributable CVD mortality, independent of age and sex.

This study sheds light on the complex relationship between physical activity and PM2.5-attributable CVD mortality, emphasizing the need for public health awareness and policy-making efforts to mitigate air pollution risks. Our findings can inform individuals' decisions about when and where to exercise, ultimately promoting cardiovascular health while minimizing air pollution exposure. Furthermore, the results highlight the importance of implementing strategies to reduce air pollution levels, ensuring a safer environment for outdoor physical activities.

Reducing air pollution and its adverse effects is crucial for improving public health and mitigating the impact of CVD. To achieve this, several key recommendations can be considered. First, promoting clean energy sources such as solar, wind, and hydroelectric power can significantly reduce air pollution from power plants and industrial facilities. Second, improving transportation options such as public transportation, carpooling, and cycling can help reduce emissions from vehicles. Stricter vehicle emission standards and the promotion of electric and hybrid vehicles are also essential. Third, enforcing strict regulations and emission standards for industries and factories can control the release of harmful pollutants into the atmosphere. Additionally, raising public awareness about indoor air pollution and encouraging the use of air purifiers and proper ventilation can improve indoor air quality. Urban planning that incorporates green spaces, parks, and tree planting can enhance air quality and provide natural air filtration. Implementing educational campaigns can promote behavioral changes to reduce exposure to air pollution. Establishing and maintaining a comprehensive air quality monitoring system is crucial for tracking pollution levels and informing the public about potential risks. Collaboration with neighboring countries to address transboundary air pollution and support for research and innovation are also vital. Furthermore, targeted interventions for vulnerable populations, such as children, the older adults, and individuals with pre-existing health conditions, should be implemented to reduce their exposure to air pollution. By incorporating these recommendations, governments, policymakers, and communities can take significant steps toward reducing air pollution and protecting public health from the adverse effects associated with cardiovascular diseases and other health problems.

The principal strength of our analysis lies in the investigation of nationwide data on PA and ambient PM2.5-attributable cardiovascular deaths, enabling the evaluation of disparities in state trends and PA prevalence. However, our study has several limitations. First, PA data were self-reported via questionnaires subject to recall bias and social desirability bias. Second, the data source lacks detailed individual clinical and risk factor information, limiting our ability to account for these factors when examining mortality trends and PA. Third, we employed an ecological approach to investigate PA prevalence and AAPC of mortality at the state level. Fourth, we acknowledge the limitation of using a simplified binary PA definition that may not capture the full spectrum of PA behaviors. Fifth, we were unable to obtain sufficient data to adjust for potential confounders such as smoking, diet, and socioeconomic status. This may have influenced the association between physical activity and cardiovascular mortality. Despite its methodological limitations, an ecological approach is the most appropriate design to address our research hypotheses. We believe that our results will be particularly helpful for state public health officials and the general public to increase awareness of PM2.5-attributable CVD and reduce CVD deaths associated with PA and PM2.5 exposure.

## 5. Conclusion

Our study reveals a positive association between PM2.5-attributable CVD mortality trends and state-level PA prevalence, with significant variations among different states. This finding suggests that PA may play a crucial role in mitigating the cardiovascular effects of air pollution. However, due to the cross-sectional design, establishing a direct causal relationship is limited. Further research is needed to establish the causal relationship.

## Data availability statement

The original contributions presented in the study are included in the article/supplementary material, further inquiries can be directed to the corresponding author.

## Author contributions

All authors listed have made a substantial, direct, and intellectual contribution to the work and approved it for publication.
